# Effects of direct oral anticoagulants dabigatran and rivaroxaban on the blood coagulation function in rabbits

**DOI:** 10.1515/biol-2022-0002

**Published:** 2022-01-28

**Authors:** Lu Yin, Yuan Qi, Zhiru Ge, Jiajin Li

**Affiliations:** Department of Cardiology, Gongli Hospital of Shanghai Pudong New Area, Shanghai 200000, China; Department of Cardiology, The First Affiliated Hospital of Soochow University, Suzhou, Jiangsu, China; Department of Nuclear Medicine, Renji Hospital, School of Medicine, Shanghai Jiao Tong University, 160 Pujian Road, Shanghai 200127, China

**Keywords:** dabigatran, rivaroxaban, anticoagulants, thrombin, APC

## Abstract

The aim of this study was to explore the effects of dabigatran and rivaroxaban on the activities of various coagulation factors. To achieve that, 60 rabbits were randomly divided into experimental groups that received different doses of dabigatran or rivaroxaban. The effects of dabigatran and rivaroxaban on the activities of FII, FV, FVIII, FX, and activated protein C (APC) were analyzed. In the dabigatran groups, activated partial thromboplastin time and thromboplastin time (TT) were prolonged after drug administration, and the activities of FII, FV, FVIII, and FX were inhibited as the drug concentration increased. Low doses of dabigatran inhibited APC activity. In the rivaroxaban groups, APTT and TT were not significantly prolonged after drug administration. In contrast, the high-dose rivaroxaban group exhibited prolonged PT, and the degree of inhibition of the activities of FII, FV, FVIII, and FX increased as the drug concentration increased. Rivaroxaban had no significant effect on APC activity regardless of dosage. As the drug concentration increased, both NOACs had more significant inhibitory effects on the activities of FII, FV, FVIII, and FX. Low concentrations of dabigatran generated an inhibitory effect on APC activity, while high concentrations of dabigatran had no significant effect. Rivaroxaban had no significant effect on APC activity.

## 1 Introduction

Atrial fibrillation (AF) is a common arrhythmia condition. Approximately, one-third of the hospitalized patients with arrhythmia have AF. In 2020, 5.6 million people in the United States suffered from AF, of which half were elderly people over 80 years of age [1]. In patients with concurrent nonvalvular atrial fibrillation (NVAF), the incidence of ischemic stroke is 6 times that of patients without NVAF [2].

Oral anticoagulation therapy is the cornerstone of stroke prevention [3]. The traditional drug warfarin has a definite curative effect [4]. However, this drug has several shortcomings. Its effect can be easily affected by food and other drugs, and it is necessary to monitor the blood coagulation function of patients. Insufficient or excessive anticoagulation may readily occur, leading to an increased risk of ischemia or hemorrhage in patients [5]. Therefore, the development of direct new oral anticoagulants (NOACs) becomes inevitable.

In clinical practice, NOACs mainly include the direct inhibitors of thrombin FII (clotting factor II), i.e., dabigatran, and the inhibitors of coagulation factor FX, i.e., rivaroxaban [6]. Although the mechanisms of action vary among different types of oral anticoagulants, the final effects are all related to the inhibition of FII. Thus, these drugs achieve anticoagulation by reducing the production of thrombin or inhibiting its activity [7].

Dabigatran and rivaroxaban are both newly developed nonvitamin K-dependent anticoagulants [7,8]. Compared with the traditional anticoagulant warfarin, these drugs have advantages such as a single target, fast effects, short half-life, no need to monitor blood coagulation, and less interaction with drugs and food [9]. At present, both oral dabigatran (the active ingredient is dabigatran) and rivaroxaban are effectively used in anticoagulation treatment for patients with AF and venous thromboembolism [10].

Although dabigatran and rivaroxaban are specific inhibitors of FII and FX, studies have found that the activities of other coagulation factors are also altered in patients who take dabigatran and rivaroxaban [11,12]. However, in the abovementioned studies, experiments were conducted *in vitro* using the blood of volunteers. Therefore, the present study examined the changes in the activities of FII, FV, FVIII, FX, and activated protein C (APC) in healthy experimental animals after the administration of various concentrations of dabigatran and rivaroxaban. The present study intended to explore whether different NOACs had different effects on thrombin activity.

## 2 Methods

### 2.1 Grouping of the research animals

A total of 60 healthy New Zealand white rabbits weighing 2.0–2.6 kg were selected for this study. The experimental animals were bred and provided by the Animal Experiment Center of Soochow University. They were divided into the dabigatran group and rivaroxaban group according to the principle of randomization. The dabigatran group was further divided into the blank control, 1, 5, 10, 20, and 40 mg/kg groups [13], with 6 animals in each group. The rivaroxaban group was divided into the blank control and the 0.3, 1, and 3 mg/kg groups [14], and each group contained six animals.


**Ethical approval:** The research related to animal use has been complied with all the relevant national regulations and institutional policies for the care and use of animals.

### 2.2 Animal experiment methods

Dabigatran capsules, with the active form of the prodrug dabigatran etexilate, were provided by Boehringer–Ingelheim. After the tablets were crushed, the stock solution was prepared in dimethyl sulfoxide (DMSO) in a volume ratio of 1:9. On the day of the experiment, the stock solution was further diluted in DMSO with different concentrations prepared according to the rabbit’s weight and grouping [15]. According to the Biopharmaceutics Classification System, Rivaroxaban is a class II drug with low solubility and high permeability. The dosage of rivaroxaban was calculated according to the weight and grouping of rabbits. After being crushed, it was slightly dissolved in water and treated by intragastric administration [16]. Before drug administration, 3 mL of blood was collected from the marginal ear vein of each animal into anticoagulant tubes. After blood was collected, the animals were immobilized, and a gavage tube was inserted into the stomach. Animals in the dabigatran groups were gavaged with dabigatran solution at concentrations of 1, 5, 10, 20, and 40 mg/kg, respectively (10 mL each time), and aminals in the blank control group were gavaged with 10 mL of solvent. Animals in the rivaroxaban groups were gavaged with rivaroxaban solution at concentrations of 0.3, 1, and 3 mg/kg respectively (10 mL each time), and animals in the blank control group were gavaged with 10 mL of distilled water. Approximately 2 h after gavage, 3% sodium pentobarbital was injected into the animals through the marginal ear vein at a dose of 1 mL/kg. After the corneal reflex disappeared, with level 1 tension in the limbs, the animals were immobilized on an animal experimental table in the supine position. Blood (3 mL) was collected from the femoral artery, transferred into anticoagulant tubes, and mixed immediately by inverting the tubes. Within 30 min of collection, blood was centrifuged at 2,500 rpm for 15 min. The plasma in the upper layer was collected and stored in −80°C freezer for future assays [17].

### 2.3 Examination of coagulation factors

The following are the methods and required reagents for assessing various indicators. The Jiangsu Institute of Hematology of the First Affiliated Hospital of Soochow University examined all the blood coagulation indicators and coagulation factor activities using a STAGO compact automatic blood coagulation analyzer (France) in accordance with the manufacturer’s instructions. All related reagents were also provided by the company [17].

The activated partial thromboplastin time (APTT), plasma prothrombin time (PT), and thrombin time (TT) were measured on a STAGO compact automatic coagulometer using a coagulation method-based APTT assay kit, PT assay kit, and TT assay kit by the manufacturer’s instructions.

#### 2.3.1 FII, FV, FVIII, and FX activities

The activities of FII, FV, FVIII, and FX were measured on a STAGO compact automatic coagulometer using coagulation method-based kits. According to the literature, Gerotziafas determined the activities of coagulation factors using a method involving various folds of dilution [10]. Therefore, we also examined the activities of FII, FV, FVIII, and FX after diluting the samples. Specifically, the measured values of the activities of FII, FV, and FVIII fell within the appropriate range after 5-, 50-, and 20-fold dilutions, respectively. Hence, the above method was used to determine the activities of the coagulation factors.

#### 2.3.2 APC activity

APC activity was measured on a STAGO compact automatic coagulometer using an APC activity kit (chromogenic substrate method).

### 2.4 Statistical analysis

Statistical analysis was performed using the SPSS 19.0 statistical package. Measurement data are expressed as the mean ± standard deviation. Significant differences between measurement data were determined using the paired sample *t*-test. *P* values of less than 0.05, 0.01, and 0.001 indicated that the differences were statistically significant. Plots were constructed using GraphPad software. In the figures, # indicates that a difference was not statistically significant (*P* > 0.05); *P < 0.05; **P < 0.01; and ***P < 0.001.

## 3 Results

### 3.1 Effects of various doses of oral dabigatran and rivaroxaban on the coagulation function

#### 3.1.1 Dabigatran groups

**Figure 1:**
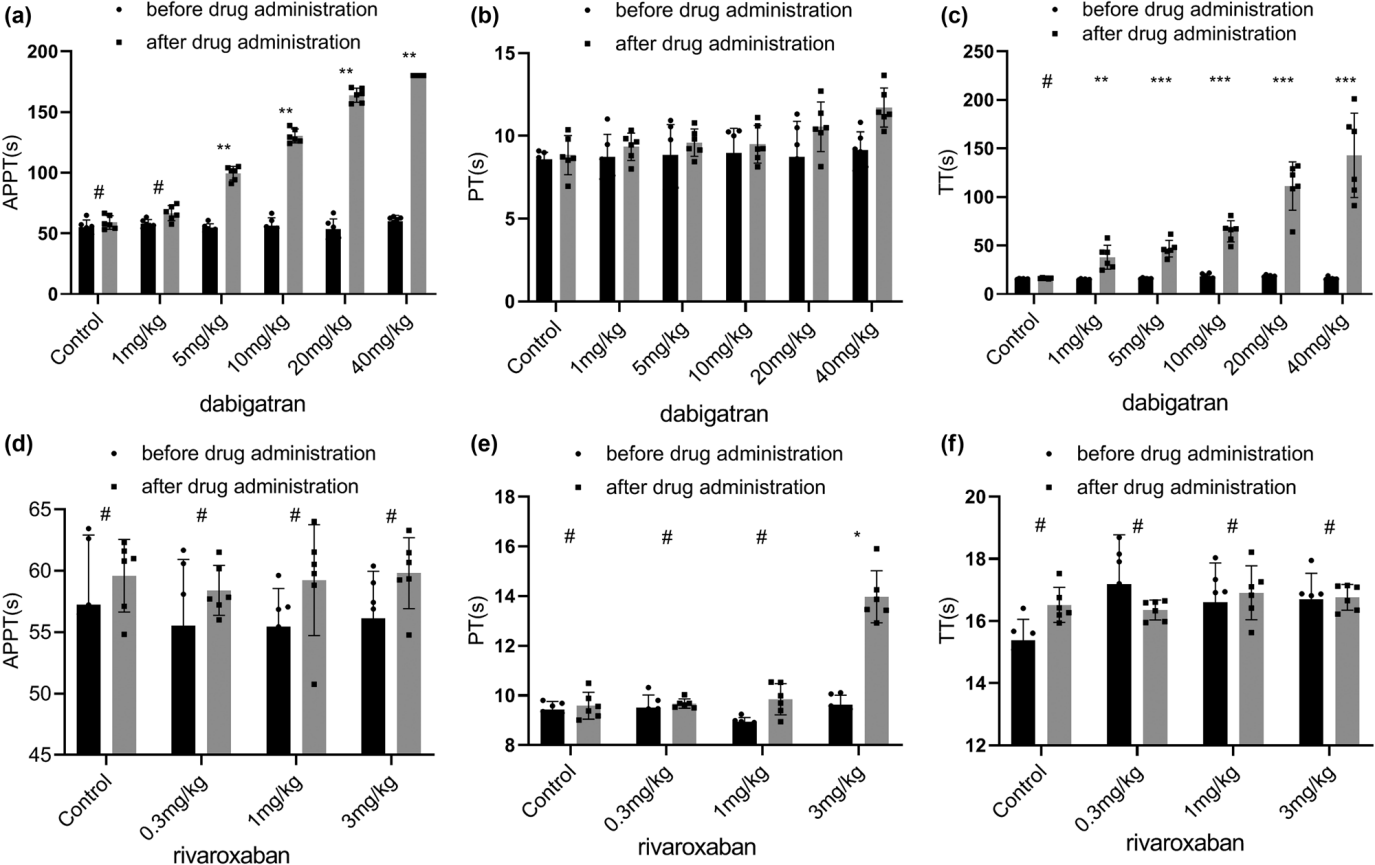
Effects of various doses of oral dabigatran and rivaroxaban on the activated partial thromboplastin time (APTT; (a) and (d)), the prothrombin time (PT; (b) and (e), and the thrombin time (TT; (c) and (f)) in rabbits. *p < 0.05, **p < 0.01, ***p < 0.001 versus the control group; (mean ± S.D., *n* = 6).

Paired *t*-tests were performed to analyze APTT, PT, and TT in each group before and after drug administration. There was no difference in the APTT between the control group and the 1 mg/kg dose group (*P* > 0.05) (Figure 1a). APTT became prolonged in the 5 mg/kg dose group, and the difference was statistically significant (*P* < 0.05). There was no difference in PT among the 1 mg/kg group, the 5 mg/kg group, and the 10 mg/kg group, suggesting that PT was not sensitive to dabigatran dose and only began to become significantly prolonged (*P* < 0.05) at a higher dose (20 mg/kg) (Figure 1b). In contrast, TT was very sensitive to dabigatran dose and became significantly prolonged in the low-dose group (1 mg/kg) (*P* < 0.01) (Figure 1c). (Note: In the 40 mg/kg group, the APTT value exceeded the upper limit of measurement (180 s), and the result was replaced with 180 s.)

#### 3.1.2 Rivaroxaban groups

After drug administration, there was no significant difference in PT values among the control group, the 0.3 mg/kg dose group, and the 1.0 mg/kg dose group (*P* = 0.322). However, the differences between the 3.0 mg/kg dose group and the other three groups were statistically significant (*P* < 0.01) (Figure 1e). There was no significant difference in the APTT level among the control group, the 0.3 mg/kg dose group, the 1.0 mg/kg dose group, and the 3 mg/kg group (*P* = 0.246) (Figure 1d). After drug administration, TT gradually became slightly prolonged in each group as the dose of rivaroxaban increased (Figure 1f). However, variance analysis indicated that the change was not statistically significant (*P* = 0.864). Rivaroxaban had no effect on TT.

### 3.2 Effects of various doses of oral dabigatran and rivaroxaban on the activities of coagulation factors

FII, FV, FVIII, FX, and APC activities were examined before and after intragastric administration of the two drugs. Changes in the activity of these factors before and after administration of the drugs were analyzed. The degree of activity change before and after administration of the drugs was analyzed, namely, the rate of change = (activity before medication-activity after medication)/activity before medication. The results indicated no significant differences in the activities of FII, FV, FVIII, FX, and APC in the control group before and after treatment (*P* > 0.05).

**Figure 2:**
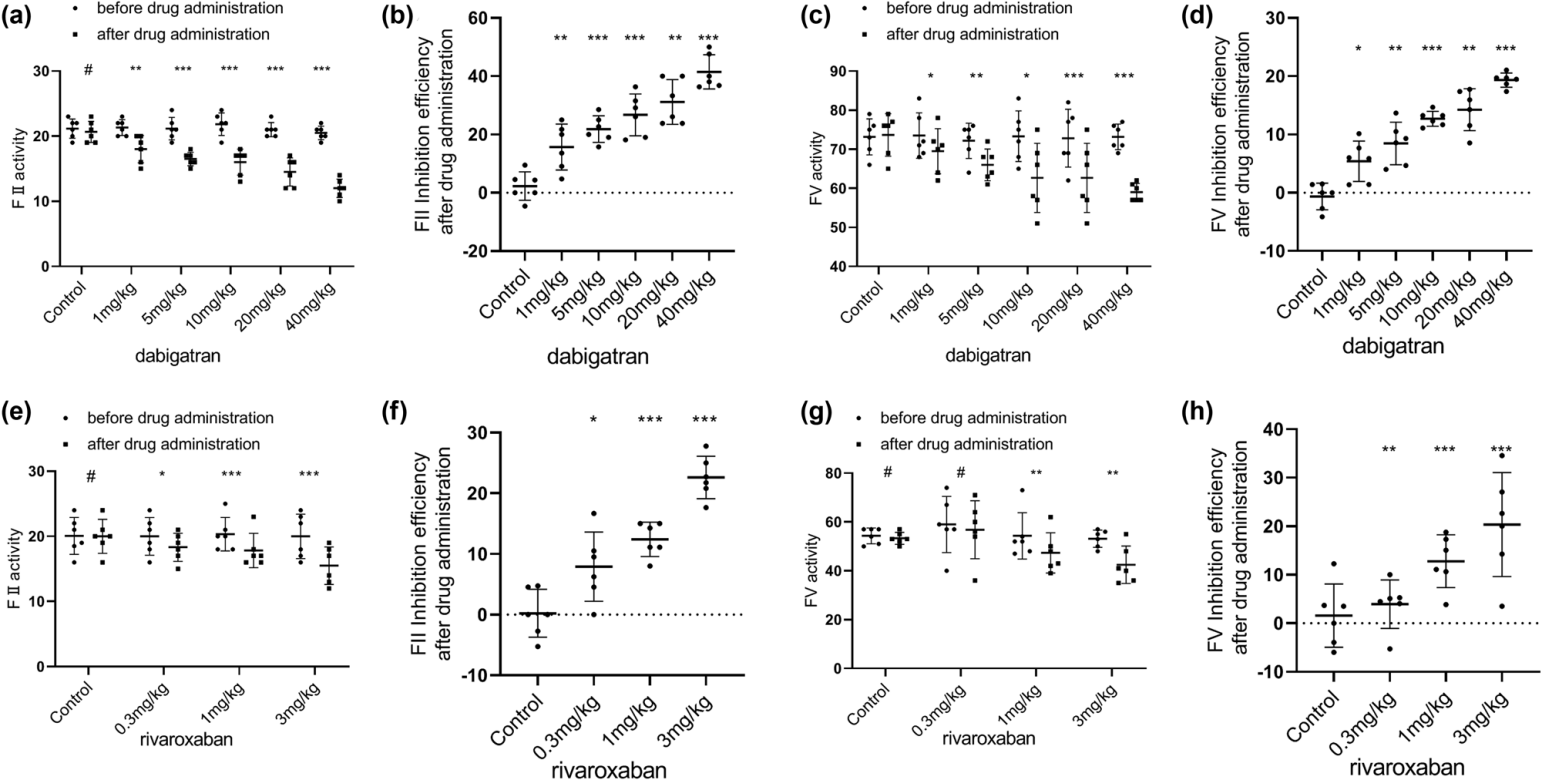
Effects of various doses of oral dabigatran and rivaroxaban on coagulation factor 2 (FII; (a) and (e)), 5 (FV; (c) and (g)) activity in rabbits. (b) and (d) The rate of inhibition of FII and FV activity by dabigatran. (f and h) The rate of inhibition of FII and FV activity by rivaroxaban. *p < 0.05, **p < 0.01, ***p < 0.001 versus the control group; (mean ± S.D., *n* = 6).

In all groups, FII activity decreased after drug administration compared with that before drug administration. Among the dabigatran groups, the decrease in FII activity was statistically significant at 1 mg/kg (*P* < 0.01). From 5 to 40 mg/kg, FII activity was markedly reduced and the decrease was also statistically significant (*P* < 0.001) (Figure 2a). Among the rivaroxaban groups, the decrease in FII activity was statistically significant at 0.3 mg/kg (*P* < 0.05), although it was more significant in the 1 mg/kg group and the 3 mg/kg group (*P* < 0.01) (Figure 2e). Regarding the rate of inhibition of FII activity, the dabigatran groups exhibited an inhibition efficiency of 15.7% at 1 mg/kg and 41.4% at 40 mg/kg (Figure 2b). The rivaroxaban groups had an inhibition efficiency of 7.9% at 0.3 mg/kg and 22.6% at 3 mg/kg (Figure 2f).

In all groups, FV activity was reduced after drug administration compared with that before drug administration. Among the dabigatran groups, the decrease in FV activity was statistically significant at 1 mg/kg (*P* < 0.05). From 5 mg/kg to 40 mg/kg, the decrease in FV activity was also significant (*P* < 0.01) (Figure 2c). Among the rivaroxaban groups, the 0.3 mg/kg group exhibited no significant difference in FV activity before and after drug administration (*P* > 0.05) (Figure 2g). However, FV activity was significantly decreased in the 1 and 3 mg/kg groups after drug administration (*P* < 0.01). Regarding the rate of inhibition of FV activity, the dabigatran groups exhibited an inhibition efficiency of 5.4% at 1 mg/kg and 19.2% at 40 mg/kg (Figure 2d). The rivaroxaban groups exhibited an inhibition efficiency of 3.9% at 0.3 mg/kg and 20.3% at 3 mg/kg (Figure 2h).

**Figure 3:**
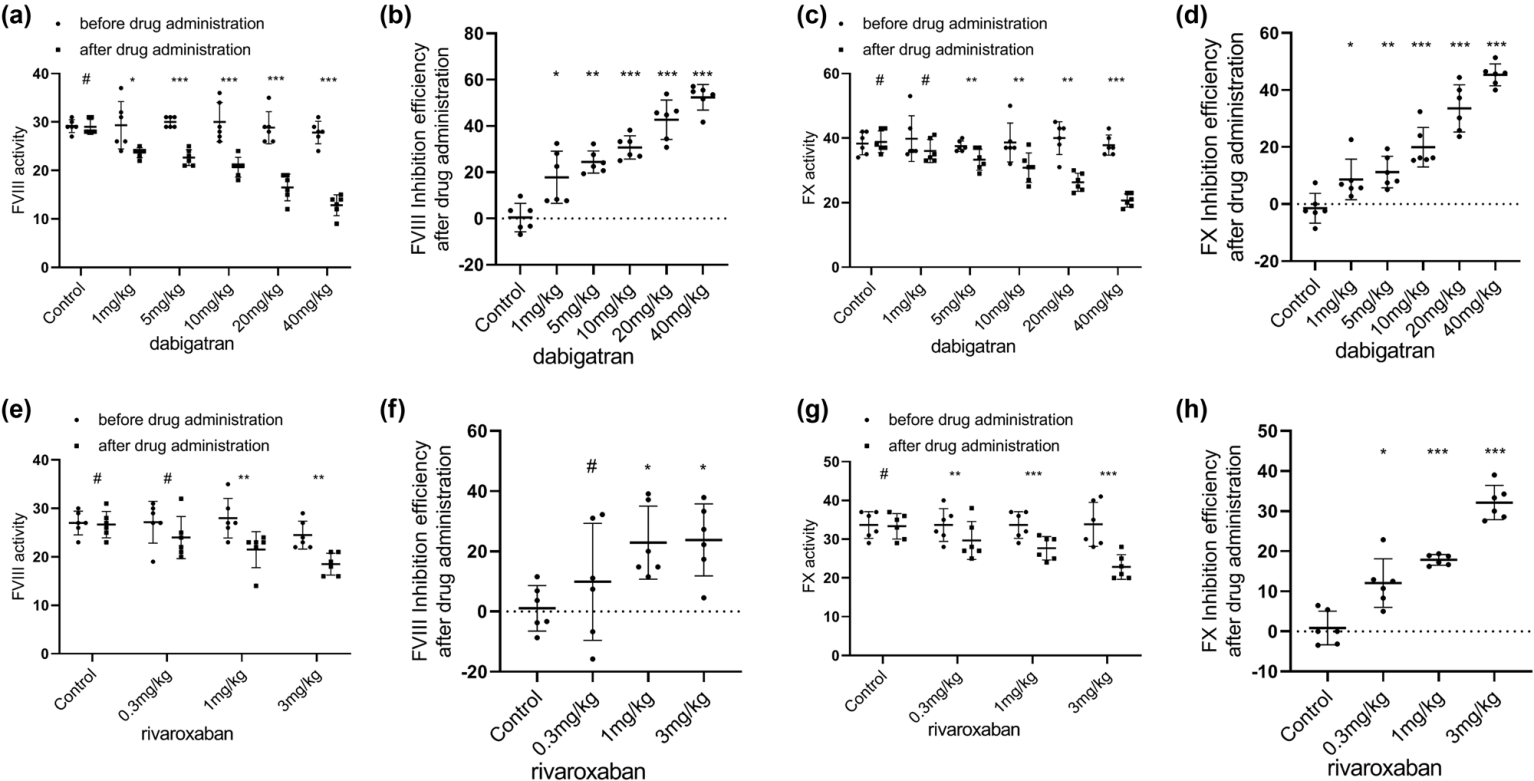
Effects of various doses of oral dabigatran and rivaroxaban on coagulation factor 8 (FVIII; (a) and (e)), 10 (FX; (c) and (g)) activity in rabbits. (b) and (d) The rate of inhibition of FII and FV activity by dabigatran. (f) and (h) The rate of inhibition of FII and FV activity by rivaroxaban. *p < 0.05, **p < 0.01,***p < 0.001 versus the control group; (mean ± S.D., *n* = 6).

In all groups, FVIII activity was decreased after drug administration compared with that before drug administration. Among the dabigatran groups, the decrease in FVIII activity was statistically significant at 1 mg/kg (*P* < 0.05). From the 5 mg/kg group to the 40 mg/kg group, FVIII activity was markedly reduced, and the decrease was also statistically significant (*P* < 0.001) (Figure 3a). In the group treated with 0.3 mg/kg rivaroxaban, FVIII activity did not change significantly (*P* > 0.05). However, FVIII activity significantly decreased in the 1 mg/kg group and the 3 mg/kg group (*P* < 0.01) (Figure 3e). Regarding the rate of inhibition of FV activity, the dabigatran groups exhibited an inhibition efficiency of 17.8% at 1 mg/kg and 52.4% at 40 mg/kg (Figure 3b). The rivaroxaban groups exhibited an inhibition efficiency of 9.8% at 0.3 mg/kg and 23.7% at 3 mg/kg (Figure 3f).

In all groups, FX activity decreased after drug administration compared with that before drug administration. Among the dabigatran groups, FX activity did not change significantly in the 1 mg/kg group. The decrease in FX activity was statistically significant at 5 mg/kg (*P* < 0.01). From the 5 mg/kg group to the 40 mg/kg group, FX activity was reduced, and the reduction was also statistically significant (*P* < 0.001) (Figure 3c). In the group treated with 0.3 mg/kg rivaroxaban, FX activity decreased considerably, and the decrease was statistically significant (*P* < 0.05) (Figure 3g). FX activity was also significantly reduced in the 1 mg/kg group and the 3 mg/kg group (*P* < 0.01). Regarding the rate of inhibition of FX activity, the dabigatran groups exhibited an inhibition efficiency of 8.6% at 1 mg/kg and 45.3% at 40 mg/kg (Figure 3d). The rivaroxaban groups exhibited an inhibition efficiency of 12% at 0.3 mg/kg and 32.1% at 3 mg/kg (Figure 3h).

### 3.3 Effects of various doses of oral dabigatran and rivaroxaban on APC activity

**Figure 4:**
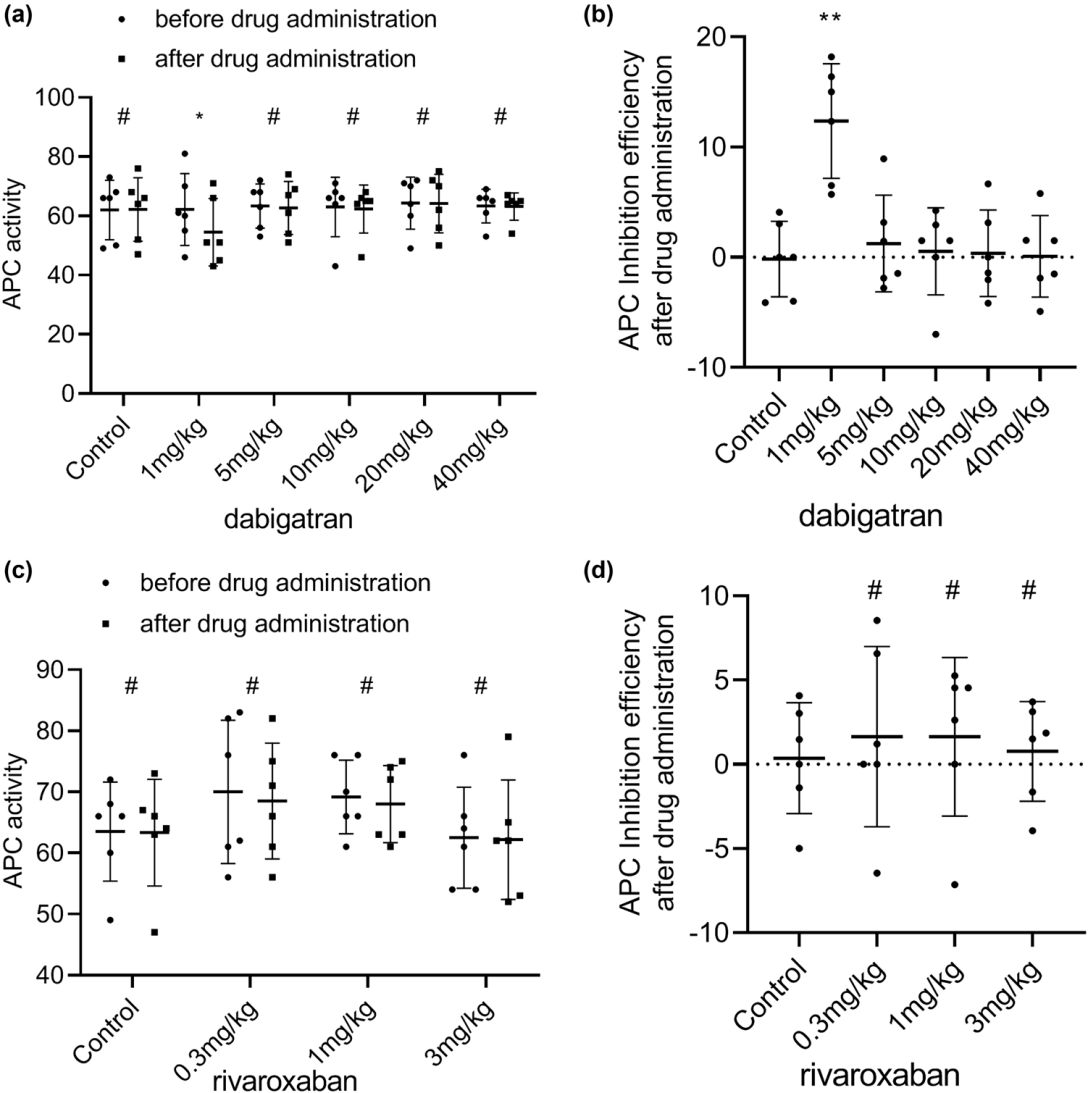
Effects of various doses of oral dabigatran and rivaroxaban on activated protein C (APC; (a) and (c)) activity in rabbits. (b) and (d) The rate of inhibition of APC activity by dabigatran and rivaroxaban, respectively. *p < 0.05, **p < 0.01, ***p < 0.001 versus the control group (mean ± S.D., *n* = 6).

APC activity exhibited a small change in all groups after drug administration compared with that before drug administration (Figure 4a). Among the dabigatran groups, APC activity was inhibited only in the 1 mg/kg group (inhibition efficiency: 12.3%) (Figure 4b), and the inhibitory effect was statistically significant (*P* < 0.05). Rivaroxaban treatment had no significant effect on the rate of inhibition of APC activity (Figures 4c and d).

## 4 Discussion

NOACs mainly include the FII inhibitor dabigatran and the FX inhibitor rivaroxaban [9]. Dabigatran etexilate itself has no activity and is a prodrug. This prodrug is metabolized by serum esterase to dabigatran after oral administration. Dabigatran binds to the fibrin-specific binding site of thrombin. As a result, fibrinogen cannot be converted into fibrin, preventing thrombus formation [18]. Rivaroxaban is a specific inhibitor of factor X. It reaches peak plasma concentration 2–4 h after oral administration. The half-life of rivaroxaban is 9–12 h, and 40% of rivaroxaban is metabolized through the kidneys [19].

To explore the effects of dabigatran and rivaroxaban on the activities of FII and FX, the present study treated healthy rabbits with various concentrations of dabigatran and rivaroxaban and examined the activities of FII and FX before and after treatment. The results showed that the activity of FII decreased as the concentration of dabigatran increased. When the dabigatran concentration was greater than or equal to 10 mg/kg, the decrease in FII activity reached a statistically significant level. Moreover, as the concentration increased, the rate of change increased accordingly. Such results indicate that the degree of FII activity inhibition was increasing. In the experimental groups that received rivaroxaban, FX activity decreased considerably as the rivaroxaban concentration increased. They reached a statistically significant level at 1 mg/kg rivaroxaban. These results confirmed that dabigatran and rivaroxaban effectively inhibited the activity of FII and FX, respectively, in healthy animals.

FX represents the point of convergence of the endogenous and exogenous coagulation pathways. FX forms a prothrombinase complex with factor V, Ca^2+^, and platelet membrane phospholipids, activating thrombin. One molecule of FX can produce 1,000 molecules of FII. Dabigatran inhibits thrombin activity by directly binding to free or bound thrombin, directly blocking the physiological function of thrombin. In addition, dabigatran also blocks the self-activation of thrombin, further reducing thrombin generation [17,20]. Rivaroxaban, an FXa inhibitor, reduces thrombin generation by inhibiting the formation of the upstream prothrombinase complex [21,22]. Anticoagulant assays of rabbit plasma indicated that both dabigatran and rivaroxaban effectively reduced the activities of FII, FV, FVIII, and FX. NOACs inhibit FII activity and reduce the positive feedback activation of FV and FVIII, thus ultimately reducing the activation of fibrinogen [7]. However, the difference in the anticoagulant mechanisms of the two types of drugs requires further investigation.

Although there is no need to monitor the coagulation function when using dabigatran and rivaroxaban, the examination of coagulation function becomes necessary under some special conditions, such as bleeding, abnormal renal function, chronic liver disease-related abnormal coagulation function, emergency surgery, and suspected drug overdose [23]. Cuker et al. found that APTT and plasma PT were not sensitive to dabigatran [24]. APTT was prolonged more than 2-fold only when the blood concentration of dabigatran was at least 400 ng/mL [25]. Analyses of APTT in AF patients receiving dabigatran indicated that APTT might still be in the normal range even if the concentration of dabigatran was 60 ng/mL. In this study, both APTT and TT became prolonged in the dabigatran groups as the dabigatran concentration increased. The prolongation of APTT and PT was not statistically significant in the 1 mg/kg group. APTT and PT started to become significantly prolonged in the 5 mg/kg group. In the 10 mg/kg group, APTT was prolonged more than 2-fold. In the 40 mg/kg group, APTT reached the upper limit of the detection range. The APTT values of the six rabbits in the 40 mg/kg group were all 180 s, indicating that the detection of APTT and PT was not accurate in the dabigatran group. At present, the more reliable indicators for detecting the activity of dabigatran are the diluted thrombin time and the Russell viper venom clotting time [25]. Regarding FX inhibitors, studies have found that compared with APTT, PT has a better linear relationship with rivaroxaban concentration and anti-FX activity [26,27]. In this study, PT was significantly prolonged in the 0.3 mg/kg rivaroxaban group.

Studies have found that warfarin has an inhibitory effect on vitamin K epoxide reductase and that it has an inhibitory effect on the production of protein C at the initial stage of use, potentially leading to a transient hypercoagulable state and an increased risk of thrombosis [28,29]. Warfarin-caused skin necrosis was also considered to be related to a decrease in protein C [18]. In addition, a study found that the direct thrombin inhibitors ximelagatran and dabigatran increased the incidence of acute coronary events such as myocardial infarction and that a high percentage of these acute coronary syndrome events occurred in the low-dose treatment group [30]. At present, there is no exact mechanism described for this phenomenon. One possible explanation is that warfarin has an antithrombotic effect more potent than that of dabigatran. In addition, damage to the APC system may also be an influencing factor [31]. Recent studies found that blocking APC (through the APC antibody SPC-54) reduced the hemorrhage caused by the FX inhibitor rivaroxaban. However, SPC-54 had no effect on the hemorrhage caused by dabigatran [32]. This finding indicates that FX and FII inhibitors cause hemorrhaging through different mechanisms [33].

This study has limitations. Certain operations such as exposing the femoral artery and drawing blood might slightly activate the coagulation system, resulting in the activation of some coagulation factors. Due to limitations related to the experimental conditions and time, the control variable in this study was drug concentration. The difference in drug absorption among the rabbits was ignored, rendering the study slightly less rigorous. However, the accurate measurement of rivaroxaban concentration by liquid chromatography-mass spectrometry cannot be achieved in clinical practice. Therefore, this study closely reflected situations observed in clinical practice.

## 5 Conclusion

Both NOACs had inhibitory effects on the activities of FII, FV, FVIII, and FX, and the inhibitory effect was more significant as the drug concentration increased. Low concentrations of dabigatran generated an inhibitory effect on APC activity, while high concentrations of dabigatran had no significant effect. Rivaroxaban had no significant effect on APC activity.

## References

[1] Sozener CB, Lisabeth LD, Shafie-Khorassani F, Kim S, Zahuranec DB, Brown DL, et al. Trends in stroke recurrence in Mexican americans and non-Hispanic whites. Stroke. 2020;51:2428–34. 10.1161/STROKEAHA.120.029376 PMC7387185 32673520

[2] Hannon N, Sheehan O, Kelly L, Marnane M, Merwick A, Moore A, et al. Stroke associated with atrial fibrillation – incidence and early outcomes in the north Dublin population stroke study. Cerebrovasc Dis. 2010;29:43–9. 10.1159/000255973 PMC2914401 19893311

[3] Kotalczyk A, Mazurek M, Kalarus Z, Potpara TS, Lip GYH. Stroke prevention strategies in high-risk patients with atrial fibrillation. Nat Rev Cardiol. 2021;18:276–90. 10.1038/s41569-020-00459-3 33110242

[4] Weitz JI, Chan NC. Novel antithrombotic strategies for treatment of venous thromboembolism. Blood. 2020;135:351–9. 10.1182/blood.2019000919 31917385

[5] Wozakowska-Kaplon B, Bartkowiak R, Grabowska U, Janiszewska G. Persistent atrial fibrillation is not associated with thrombomodulin level increase in efficiently anticoagulated patients. Arch Med Sci. 2010;6:887–91. 10.5114/aoms.2010.19297 PMC3302700 22427762

[6] Tripodi A. The laboratory and the direct oral anticoagulants. Blood. 2013;121:4032–5. 10.1182/blood-2012-12-453076 23564912

[7] Polzin A, Dannenberg L, Wolff G, Helten C, Achilles A, Hohlfeld T, et al. Non-vitamin K oral anticoagulants (NOAC) and the risk of myocardial infarction: differences between factor IIa and factor Xa inhibition? Pharmacol Ther. 2019;195:1–4. 10.1016/j.pharmthera.2018.10.005 30321554

[8] Xu W, Lv M, Wu S, Jiang S, Zeng Z, Fang Z, et al. Severe bleeding risk of direct oral anticoagulants versus vitamin k antagonists for stroke prevention and treatment in patients with atrial fibrillation: a systematic review and network meta-analysis. Cardiovasc Drugs Ther. 2021. 10.1007/s10557-021-07232-9. 34436708

[9] Chan N, Sobieraj-Teague M, Eikelboom JW. Direct oral anticoagulants: evidence and unresolved issues. Lancet. 2020;396:1767–76. 10.1016/S0140-6736(20)32439-9 33248499

[10] López-López JA, Sterne JAC, Thom HHZ, Higgins JPT, Hingorani AD, Okoli GN, et al. Oral anticoagulants for prevention of stroke in atrial fibrillation: systematic review, network meta-analysis, and cost effectiveness analysis. BMJ. 2017;359:j5058. 10.1136/bmj.j5058 PMC5704695 29183961

[11] Tsutsumi Y, Shimono J, Ohhigashi H, Ito S, Shiratori S, Teshima T. Analysis of the influence of dabigatran on coagulation factors and inhibitors. Int J Lab Hematol. 2015;37:225–30. 10.1111/ijlh.12270 24963788

[12] Gerotziafas GT, Baccouche H, Sassi M, Galea V, Chaari M, Hatmi M, et al. Optimisation of the assays for the measurement of clotting factor activity in the presence of rivaroxaban. Thromb Res. 2012;129:101–3. 10.1016/j.thromres.2011.09.004 22000405

[13] Wienen W, Stassen JM, Priepke H, Ries UJ, Hauel N. Antithrombotic and anticoagulant effects of the direct thrombin inhibitor dabigatran, and its oral prodrug, dabigatran etexilate, in a rabbit model of venous thrombosis. J Thromb Haemost. 2007;5:1237–42. 10.1111/j.1538-7836.2007.02526.x 17362226

[14] Perzborn E, Heitmeier S, Buetehorn U, Laux V. Direct thrombin inhibitors, but not the direct factor Xa inhibitor rivaroxaban, increase tissue factor-induced hypercoagulability in vitro and in vivo. J Thromb Haemost. 2014;12:1054–65. 10.1111/jth.12591 PMC4285304 24766850

[15] Arellano-Rodrigo E, Fernandez-Gallego V, López-Vilchez I, Molina P, Díaz-Ricart M, Zafar MU, et al. Idarucizumab, but not procoagulant concentrates, fully restores dabigatran-altered platelet and fibrin components of hemostasis. Transfusion. 2019;59:2436–45. 10.1111/trf.15259 30946491

[16] Elsayad MK, Mowafy HA, Zaky AA, Samy AM. Chitosan caged liposomes for improving oral bioavailability of rivaroxaban: in vitro and in vivo evaluation. Pharm Dev Technol. 2021;26:316–27. 10.1080/10837450.2020.1870237 33356742

[17] Zhang C, Zhang P, Li H, Han L, Zhang L, Zhang L, et al. The effect of dabigatran on thrombin generation and coagulation assays in rabbit and human plasma. Thromb Res. 2018;165:38–43. 10.1016/j.thromres.2018.03.012 29558660

[18] Stangier J. Clinical pharmacokinetics and pharmacodynamics of the oral direct thrombin inhibitor dabigatran etexilate. Clin Pharmacokinet. 2008;47:285–95. 10.2165/00003088-200847050-00001 18399711

[19] Turpie AG, Kreutz R, Llau J, Norrving B, Haas S. Management consensus guidance for the use of rivaroxaban – an oral, direct factor Xa inhibitor. Thromb Haemost. 2012;108:876–86. 10.1160/TH12-03-0209 23014816

[20] Bloemen S, Zwaveling S, Douxfils J, Roest M, Kremers R, Mullier F. The anticoagulant effect of dabigatran is reflected in the lag time and time-to-peak, but not in the endogenous thrombin potential or peak, of thrombin generation. Thromb Res. 2018;171:160–6. 10.1016/j.thromres.2018.10.005 30316961

[21] Liu Z, Xie Q, Zhang H, Mu G, Zhou S, Wang Z, et al. Target drug-calibrated anti-Xa activity assays and expected peak-trough levels in an Asian population: a multicenter study. Am J Cardiovasc Drugs. 2021;21:669–679. 10.1007/s40256-021-00479-5 34142346

[22] Abraham NS, Castillo DL. Novel anticoagulants: bleeding risk and management strategies. Curr Opin Gastroenterol. 2013;29:676–83. 10.1097/MOG.0b013e328365d415 24100724

[23] Levy JH, Douketis J, Weitz JI. Reversal agents for non-vitamin K antagonist oral anticoagulants. Nat Rev Cardiol. 2018;15:273–81. 10.1038/nrcardio.2017.223 29345686

[24] Cuker A, Siegal DM, Crowther MA, Garcia DA. Laboratory measurement of the anticoagulant activity of the non-vitamin K oral anticoagulants. J Am Coll Cardiol. 2014;64:1128–39. 10.1016/j.jacc.2014.05.065 PMC4167772 25212648

[25] Harenberg J, Giese C, Marx S, Kramer R. Determination of dabigatran in human plasma samples. Semin Thromb Hemost. 2012;38:16–22. 10.1055/s-0031-1300947 22314599

[26] Huisman MV, Lip GY, Diener HC, Brueckmann M, van Ryn J, Clemens A. Dabigatran etexilate for stroke prevention in patients with atrial fibrillation: resolving uncertainties in routine practice. Thromb Haemost. 2012;107:838–47. 10.1160/TH11-10-0718 22318514

[27] Harenberg J, Marx S, Kramer R, Giese C, Weiss C. Determination of an international sensitivity index of thromboplastin reagents using a WHO thromboplastin as calibrator for plasma spiked with rivaroxaban. Blood Coagul Fibrinolysis. 2011;22:637–41. 10.1097/MBC.0b013e328349f1d6 21986465

[28] Connolly SJ, Pogue J, Eikelboom J, Flaker G, Commerford P, Franzosi MG, et al. Benefit of oral anticoagulant over antiplatelet therapy in atrial fibrillation depends on the quality of international normalized ratio control achieved by centers and countries as measured by time in therapeutic range. Circulation. 2008;118:2029–37. 10.1161/CIRCULATIONAHA.107.750000 18955670

[29] Camm AJ, Lip GY, De Caterina R, Savelieva I, Atar D, Hohnloser SH, et al. 2012 focused update of the ESC guidelines for the management of atrial fibrillation: an update of the 2010 ESC Guidelines for the management of atrial fibrillation – developed with the special contribution of the European Heart Rhythm Association. Europace. 2012;14:1385–413. 10.1093/europace/eus305 22923145

[30] Eriksson BI, Quinlan DJ, Weitz JI. Comparative pharmacodynamics and pharmacokinetics of oral direct thrombin and factor xa inhibitors in development. Clin Pharmacokinet. 2009;48:1–22. 10.2165/0003088-200948010-00001 19071881

[31] Stangier J, Rathgen K, Stahle H, Reseski K, Kornicke T, Roth W. Coadministration of dabigatran etexilate and atorvastatin: assessment of potential impact on pharmacokinetics and pharmacodynamics. Am J Cardiovasc Drugs. 2009;9:59–68. 10.1007/BF03256595 19178132

[32] von Drygalski A, Bhat V, Gale AJ, Averell PM, Cramer TJ, Elias DJ, et al. An engineered factor Va prevents bleeding induced by direct-acting oral anticoagulants by different mechanisms. Blood Adv. 2020;4:3716–27. 10.1182/bloodadvances.2020001699 PMC7422119 32777068

[33] Exner T, Rigano J, Favaloro EJ. The effect of DOACs on laboratory tests and their removal by activated carbon to limit interference in functional assays. Int J Lab Hematol. 2020;42(Suppl 1):41–8. 10.1111/ijlh.13196 32543072

